# The Cardiac Centre Shisong organizes an open door day in Hilton Hotel Yaounde

**DOI:** 10.11604/pamj.2016.25.87.10191

**Published:** 2016-10-17

**Authors:** Tantchou Tchoumi Jacques Cabral, Appolonia Budzee, Ambassa Jean Claude, Fanka Marcel, Mbidzenyuy Julius Peter, Ngoran Henry

**Affiliations:** 1 St. Elizabeth Catholic General Hospital, Cardiac Centre Shisong, Kumbo, Cameroon

**Keywords:** Cardiac centre Shisong, Minister of Public Health, Cardiac congress

## Special feature

Initiated in 2002, the Cardiac Centre whose overall objective is to reduce the incidence of cardiovascular diseases (CVD) by providing quality low cost treatment to patients was inaugurated in November 19, 2009 by the Minister of Public Health, André MAMA FOUDA. As personal representative of H.E. Paul Biya, the Minister of Public Health on that same day declared the Cardiac Centre (CC) a National Referral Centre for Cardiovascular Disease [1]. Today, it remains the lone cardio-surgical centre in the Central African Sub-regions. It covers a surface area of 35,000 meters square, having a capacity of 79 beds, two intensive care units, 2 theatres, a catheterization laboratory and competent technical department which permanently takes care of all the equipment on the site. The Centre is the fruit of the collaboration amongst the Tertiary Sisters of St. Francis Cameroon and two Italian non-governmental organizations, namely, Associazione Cuore Fratello and Associazione Bambini Cardiopatici nel Mondo [2].

### Objectives of the March 2016 Forum

To create awareness and sensitize the Cameroon population and that of the Central African Sub Region on the activities of the CC; To share information, successes and challenges; To show-case the fruit of collaboration which can exist among persons with different works of life; To get feedback from the population on the impact of the CC since creation; To extend the Shisong CC family to potential collaborators and partners.

The Cameroon Cardiac Society and the Shisong CC have enjoyed long standing relationship and collaboration since the creation of the latter. In many occasions, the Society has acted as the official voice of the Center and as a protective shield in some stressful and confusing moments. Choosing to organize this story-making event during the cardiologist conference falls within the above stated reasons and objectives. This means that the participants can use one stone to shoot two birds: attending the medical forum and the CC event. More, the “open door day” and the conference fall within the same story line with the same objectives of sharing our concrete scientific experiences in this socio-geographical setup and canvassing for collaborators.

**Figure 1:**
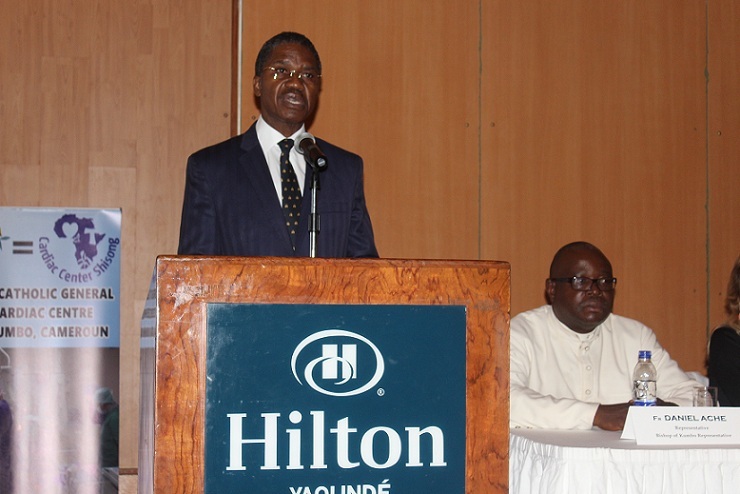
The Minister of Public Health of Cameroon his Excellency Andre Mama Fouda

**Figure 2:**
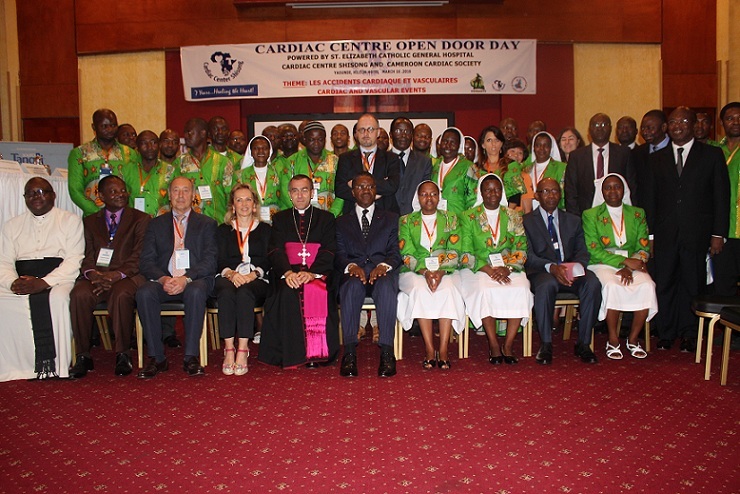
The family picture of the doctors and staff of the Cardiac Centre with the Minister of Public health

The Forum started the 16^th^ March 2016 at 8.30 am in the Bouma hall of Hilton Hotel Yaoundé, under the auspices of the Apostolic Nuncio to Cameroon and Equatorial Guinea and the Director of St. Elizabeth Catholic General Hospital Shisong, of which the Cardiac Center is a department. The Master of Ceremony, Dr. Tantchou Cabral heartily welcomed participants and invited Sr. Appolonia Budzee to say an opening prayer. The program was divided into three panels: medical, nursing, national and international Impact, whereby all the presentations were focused on the activities of the CC for the past 7-year experience. The first presentation given by Dr. Ambassa Jean Claude enlightened the audience about how CVD are managed in the Centre; the main disease being hypertension, followed by congestive heart failure and arrhythmias [3]. The General Manager, Sr. Jethro Nkenglefac, gave a keynote presentation of the Cardiac Centre, underlining the following statistics since its inception: consultations: 34,179, open heart surgery, 553; interventional and diagnostic catheterization, 379; pace makers implants, 124; intracardiac cardioverter defibrillator (ICD), 3; cardiac resynchronization therapy (CRT-P)-2’ consultations at mobile clinics, 9.963. His Excellency Andre MAMA FOUDA, Cameroon’s Minister of Public Health (Figure 1) in his inaugural speech, congratulated the administration, the doctors and the staff of the CC for their hard work, recognized the impact of the Centre on the life of Cameroonians living with cardiac problems in need of invasive procedures (Figure 2), and called on all to believe in and make use of the service instead of struggling to go overseas to get what is available at home. As Founding Partners, the President of the Italian non-governmental organization, Associazione Bambini Cardiopatici nel Mondo unveiled to the public the plans of transforming the Cardiac Centre into a teaching centre for Cameroonians and doctors of the Sub Region in invasive cardiology. To this effect, the necessary infrastructural preparations are already being mobilized. In the name of Cameroon’s Ministry of Public Health, Dr. Eyong Efobi John considered that the partnership needed to become more dynamic and fruitful. The forum, attended by 165 medics, Government officials, Diplomats, Stakeholders, journalists, NGOs, opinion leaders and Faith based organizations with national and international portforlio, ended at 13.30 pm with different interviews of the participants. Considering the cream of personalities who participated actively as the Cardiac Center staff shared their 7-year experience of healing lives, we can consider the objectives of the event met to a large extent. It was a foretaste of the celebration of the 10^th^ Anniversary which is envisaged to expose more fruitfulness, dynamism and potentials of the Cardiac Center; which is not only a health service institution, but also a platform for transcultural collaboration and icon of healthcare excellence in the Cameroonian healthcare system on the road to development.
